# Mechanical Evaluation of Suture Docking Method Versus Novel Tensegrity Suture Screw in Treating Varus Posterolateral Surrogate and Cadaveric Elbow Instability

**DOI:** 10.1111/os.70216

**Published:** 2026-01-05

**Authors:** Marilyn Janice Oentaryo, Tsz Ying Abby Yeung, Christian Fang

**Affiliations:** ^1^ Department of Orthopaedics and Traumatology The University of Hong Kong Pok Fu Lam Hong Kong China

**Keywords:** docking, elbow instability, fracture, tensegrity

## Abstract

**Introduction:**

Elbow instability often arises from collateral ligament (LUCL) complex failure, causing varus and posterolateral rotatory subluxations. Conventional docking repair requires technical expertise balancing slack and tension. Existing suture anchors lack adjustable tensioning and rely on bone tunnel length for mechanical performance. A novel tensegrity‐based suture anchor system was developed to enhance implant‐bone fixation and optimize suture tensioning for early stability.

**Methodology:**

Static three‐point bending tests were conducted using Sawbones (*n* = 20) and paired cadaveric elbows (*n* = 14) with either conventional suture docking (CON) or elbow tensegrity screw (TEN). Force‐displacement relationships were plotted. Stiffness, maximum force, and displacement at peak force were measured and compared using nonparametric Mann–Whitney *U* tests in GraphPad Prism 10.5.0.

**Results:**

In foam elbows, TEN demonstrated significantly higher stiffness (3.46 ± 1.44 N/mm) than CON (1.44 ± 1.17 N/mm, *p* < 0.01). Maximum forces were 132.60 ± 28.82 N for TEN versus 75.02 ± 20.28 N for CON (*p* < 0.01), while displacement at peak force was slightly lower in TEN (35.54 ± 5.80 mm) versus CON (39.03 ± 9.05 mm, *p* = 0.22). In cadaveric elbows, TEN also had greater stiffness (12.79 ± 9.73 N/mm) versus CON (3.53 ± 2.43 N/mm, *p* < 0.05). Maximum forces were significantly greater for TEN (199.93 ± 35.89 N) compared to CON (140.11 ± 37.23 N, *p* < 0.05), while displacements at peak force were lower in TEN (22.31 ± 10.06 mm) than CON (38.68 ± 8.64 mm, *p* < 0.05). All CON samples failed from irreversible yielding and suture stretching, whereas most TEN samples failed due to suture rupture, suggesting superior bone‐implant and suture‐implant interface resistance.

**Conclusion:**

TEN devices significantly improved mechanical strength and pretensioning over conventional docking, enhancing early stability and reducing yield failure risk.

## Graphical abstract

Three‐point bending tests in foam and cadaveric elbows consistently showed superior performance of novel TEN elbow screws in restoring elbow instability in contrast to CON docking method. TEN increased mechanical strength, enhanced suture‐screw‐bone fixation via appropriate junctional loading, and technical ease for suture tensioning.

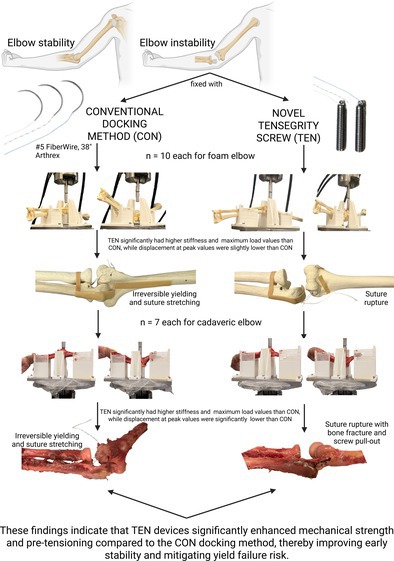

## 1 Introduction

### 1.1 Elbow Instability

The elbow joint is the second most commonly dislocated region of the upper extremity [1, 2, 3], with an incidence of 5.5 (95% CI: 4.9–6.2) per 100,000 person‐years in adults [1]. Elbow dislocation can result in chronic instability due to compromised ligamentous healing [2]. Among which, posterolateral rotatory instability (PLRI) is the dominant mechanism where the forearm externally rotates relative to the trochlea with tear of the posterolateral elbow capsular ligaments [2, 3, 4].

In the elbow, bone articulation, capsular ligaments, and muscles [4] contribute to stability. The ligamentous complex can be categorized into medial and lateral compartments, acting as main restraints. The lateral ulnar collateral ligament (LUCL) within the lateral collateral ligament (LCL) complex plays a crucial role in preventing injuries from varus stress and posterior rotatory subluxation [5]. Deficiency of the LUCL under pathological conditions is a primary cause of acute or chronic PLRI.

### 1.2 Existing Methods on Treating Elbow Instability

The primary management of acute or subacute PLRI involves reconstructing or repairing the LUCL [5, 6], which was the focus of this study. Various techniques have been developed over time to restore joint stability, albeit each with its limitations. The conventional docking technique, a common surgical approach for ligamentous reconstruction described by Jones et al., involved grafting of tendon (e.g., palmaris longus), shuttling the graft into the bony tunnel on ulnar and docking the graft to the humeral tunnel [7]. Although the graft can provide tension like the LUCL complex, it is technically demanding in precision of shuttling the sutures, tunnel placement and tensioning control; hence, positive outcomes rely greatly on the expertise of the surgeons [8, 9].

A novel technique involving suture augmentation using an internal brace for ligament repair has emerged as a promising alternative for reconstruction [10]. This technique utilizes a suture anchor loaded with a heavy braided tape suture inserted on the ulnar. The tape suture passes through the native LUCL to strengthen it. The free ends of the tape suture are shuttled into the humeral tunnel and act as the internal brace by crossing the elbow joint above the capsule and beneath the muscle group [10, 11]. However, this technique lacks the option of adjustable tensioning, and mechanical pull‐out resistance with interference screws is poor when the bone tunnel is typically shorter than 25–30 mm in the proximal ulna, especially in osteoporotic bone. The failure risk of the suture anchor's insertion is relatively high as presented by a cadaveric study [12].

In a systematic review conducted by Chang et al. [13] comparing surgical techniques for LUCL reconstruction, five out of the 17 studies investigated clinical outcomes of classic or modified docking techniques [9, 14, 15, 16, 17]. These studies reported that docking techniques achieved excellent results in at least 90% of cases with limited complications. In contrast, studies examining the Figure‐of‐8 technique (e.g., the Jobe technique) demonstrated variable rates of excellent results and complications. The docking technique has gained widespread adoption due to its superior clinical outcomes, albeit its technical complexity. Four of the seven biomechanical studies included in the review compared the maximum load and cyclic loading characteristics of docking techniques with alternative methods [18, 19, 20, 21]. Yet, the docking technique exhibited a statistically lower peak load compared to native ligaments, a finding consistent with other surgical techniques. However, alternative methods generally demonstrated even lower maximum loads, with only one study reporting a similar peak load between the Jobe and docking techniques [16]. Only one of the four studies assessed stiffness values, revealing no significant differences among the evaluated surgical techniques [19].

Although the docking technique is considered to provide good clinical outcomes, its biomechanical performance remains inferior to that of native ligaments, indicating room for improvement in treatment options which may enhance early functional rehabilitation [18, 21]. In 2019, Bernholt et al. [22] reported that the use of an internal brace showed comparable performance in maximum load and torsional stiffness compared to native ligaments, whereas the docking technique performed inferiorly. Bodendorfer et al. [23] found that internal bracing had a biomechanical profile alike to the docking technique. Despite these discrepancies, the development of new surgical techniques based on anatomical insights has led to superior performance in some cases [24]. This underscores the potential for innovative approaches, such as those leveraging the natural tensegrity system of the joint, to further advance treatment options for elbow instability.

### 1.3 Concept of Tensegrity

To address these limitations, this study introduces a novel tensegrity‐based metallic suture anchor system. Tensegrity, a term coined by mathematician Buckminster Fuller, combines “tension” and “integrity” to describe a stabilized system characterized by continuous tensile forces and discontinuous rigid components, balanced by external forces [25, 26]. In simpler terms, the tensile forces within a tensegrity system are mutually balanced, resulting in a state of equilibrium. Due to its efficient use of materials, tensegrity has been widely adopted across various fields, including engineering, architecture, and art [27]. From its esthetic applications in geometric art to its use in tensegrity soft robots in engineering and science, the concept has expanded significantly, supported by advanced mathematical tools that enhance its strength and stiffness [27, 28]. In biology, the principles of tensegrity are highly applicable for understanding structures such as the cytoskeleton (e.g., the static and dynamic nature of biconcave erythrocytes) and the musculoskeletal system (e.g., the elbow and toe joints) [27]. Given its versatility, the application of tensegrity structures holds promise for addressing pathological conditions in orthopedics. To our knowledge, this study is the first to explore a tensegrity‐based surgical technique as a treatment option, potentially inspiring future research into the therapeutic potential of tensegrity.

In the proposed surgical technique, tensegrity screws are inserted at predetermined positions and connected by FiberWire to facilitate force transmission and balance. This system offers various advantages, including tension adjustability, optimized length and diameter, enhanced implant‐bone fixation, and improved suture tensioning to achieve optimal immediate stability. FiberWire No. 5 was selected as the suture material due to its superior performance compared to conventional alternatives. A systematic review published in May 2024 demonstrated that FiberWire exhibited the highest load capacity and stiffness, along with the lowest elongation, among six commonly used suture materials [29]. This study aims to evaluate the efficacy of the novel tensegrity (TEN) system, utilizing FiberWire, in a surrogate elbow instability model for LUCL repair. Specifically, this study aimed to:

1. *Quantitatively compare the baseline biomechanical performance* of the TEN system against the conventional docking (CON) technique under controlled, reproducible conditions using a surrogate bone model, with a primary focus on stiffness, load‐to‐failure, and displacement.
2. *Validate the translational potential of the TEN system* by comparing its performance against the CON technique in a more clinically relevant cadaveric model, thereby assessing its efficacy in a biologically variable environment that simulates in vivo conditions.
3. *Characterize and interpret the failure mechanisms* of both constructs to infer their clinical failure profiles.

## 2 Material and Methods

### 2.1 Conventional Versus Tensegrity Screw in Bone Surrogate Model

**FIGURE 1:**
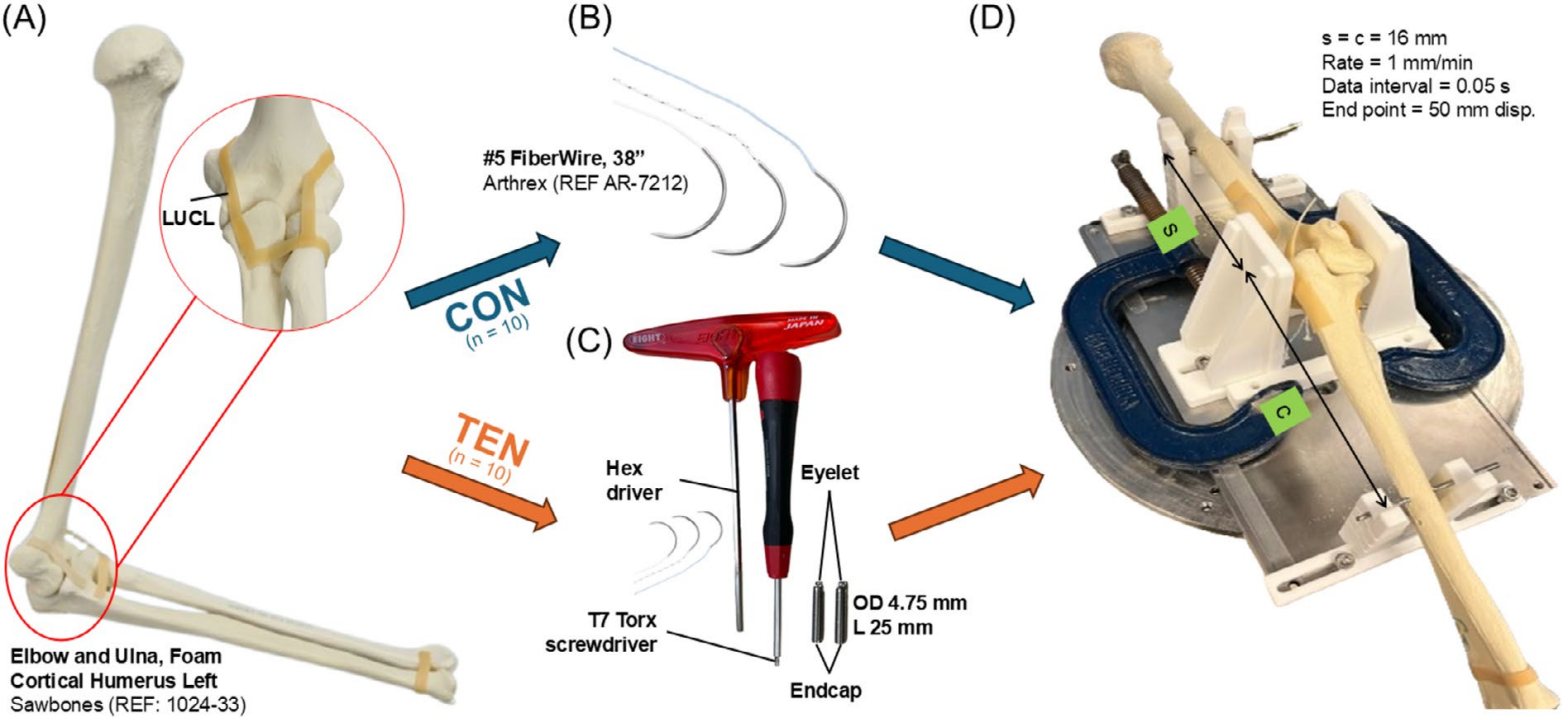
(A) Surrogate elbow and ulna, foam cortical humerus left used in the study. (B) Suture used for the conventional docking technique. (C) Instruments and elbow tensegrity screws designed and used in the study. Hex driver to insert and remove the screw, T7 Torx screwdriver to release and tension the retractable core, and the two D4.75 mm elbow tensegrity screws with retractable core with the eyelet to insert the suture and the screw endcap for the driver attachment. (D) The three‐point bending fixture consists of the stainless‐steel plate with 3D‐printed V‐shaped wedges to fix the two ends with D3 mm drill bits and 3D‐printed center angle brackets to sandwich the elbow joint construct.

Two methods were used to reconstruct the LUCL complex, either by conventional suture docking (CON) or the elbow tensegrity screw (TEN) device in left foam cortical shell elbows (REF 1024‐33, Sawbones, Figure 1A) with comparable surgical techniques (*n* = 10). High stiffness [29], FiberWire No. 5 (REF AR‐7212, Arthrex, Figure 1B) suture was used as an internal brace. All specimens, either CON or TEN, had the sutures tied with five square knots made after tensioning to ensure that loading is transferred to the bone‐implant or bone‐suture interface. This is done to eliminate suture rupture and knot slippage as the dominant mechanisms of failure.

The elbow TEN screw was specifically designed to integrate the concept of tensegrity, which is defined as a system that integrates tension and mechanical control [30]. The TEN screws had an outer diameter of 4.75 mm and a length of 25 mm, optimized for the dimensions of the elbow. Two identical screws were inserted with the hex driver into 4 mm predrilled holes at the proximal ulna LUCL tubercle and humerus lateral condyle isometric point. With the mini‐torx screwdriver, the inner set‐screw core could be withdrawn out for the suture to be fastened into an eyelet. Five square knots were then made with appropriate positioning and tensioning, which tension could be further adjusted by retracting the core using the same mini‐torx screwdriver. Tension is increased to the limit where medial side joint space opening up is observed. The maximum adjustable length for suture retraction is 5.6 mm on each side which is technically straightforward and versatile at increasing tension and eliminating slack even after suture knots were tied.

### 2.2 Power Analysis for Cadaveric Study

Performance of CON versus TEN devices in restoring the LUCL complex was further evaluated in a randomized cadaveric study. The null hypothesis posited no differences between groups in stiffness, maximum load, or displacement at maximum load, while the alternative proposed significant differences in these parameters. A power analysis based on pilot foam model data (*n* = 10 per group; *⍺* = 0.05) indicated that only two specimens per group were needed for mean load and five for stiffness to achieve 80% power. To balance cadaveric availability and implant prototyping costs, a conservative sample size of *n* = 7 per group was selected, consistent with prior biomechanical cadaveric studies [24, 31, 32, 33, 34].

### 2.3 Conventional Versus Tensegrity Screw in Cadaveric Bone Model

Baseline cadaver demographics including age, sex, race, and cause of death were recorded (refer to Appendix Table S1 in the Supporting Information). A total of 14 fresh‐frozen cadaveric elbows from Caucasian female donors with an age range from 79 to 94 years and a BMI range from 12.65 to 30.07 kg/m^2^ were studied. Donors were obtained from institutions accredited by the American Association of Tissue Banks, which accreditation requires strict adherence to standards and ensures a level of ethical practice. All specimens were treated with dignity and respect, handled by trained personnel, and disposed of properly according to institutional protocols, all while maintaining donor anonymity. The specimens were all thawed > 24 h at room temperature and all procedures of dissection, implantation and testing were done subsequently to ensure their best conditions. A standardized surgical dissection was done with all the soft tissues stripped exposing the humerus, radius, ulna and the elbow joint. All stabilizing ligaments and the elbow joint capsule were removed. Each sample had its joints dislocated for elbow instability and like the surrogate bone, the elbow sample was either restabilized with suture docking (CON) or elbow tensegrity screw (TEN). The samples were then tested for static three‐point bending.

### 2.4 Static Three‐Point Bending Test

For all the surrogate and cadaveric bone samples, three‐point bending was done with a customized testing jig machined with 316L Stainless steel and 3D‐printed PA2200 Nylon. The medial epicondyle of each sample was loaded at the midpoint of the fixture (Figure 1B) with a constant distance of 16 cm from the humeral and ulna support. Both humeral and ulna ends were rotationally fixed with diameter = 3 mm drill bits, where the elongated holes in the fixtures were designed to ensure that any lateral movement of both ends was still possible during the compression. As per ASTM F1264‐16e1 A1 test standard, static three‐point bending axial compression was performed at a rate of 1 mm/s with the MTS 858 Mini Bionix hydraulic press. The data acquisition interval was 0.05 s with an end point at 50 mm displacement. The load–displacement data were collected, and the failure mode was reported.

### 2.5 Data Analysis

From the load–displacement data, maximum load and displacement at which the maximum load was reached were obtained and compared. Meanwhile, stiffness, as the slope of the initial elastic region of the curve, was calculated at the initial displacement between the range of 0 and 5 mm. Mean and standard deviation (SD) values were calculated and summarized as the results. Statistical analyses were done for all these dependent variables with non‐parametric Mann–Whitney *U* tests (Graphpad Prism 10.5.0). Only *p*‐values < 0.05 were considered to represent statistical significance.

## 3 Results

### 3.1 Performance in Bone Surrogate Model

**FIGURE 2:**
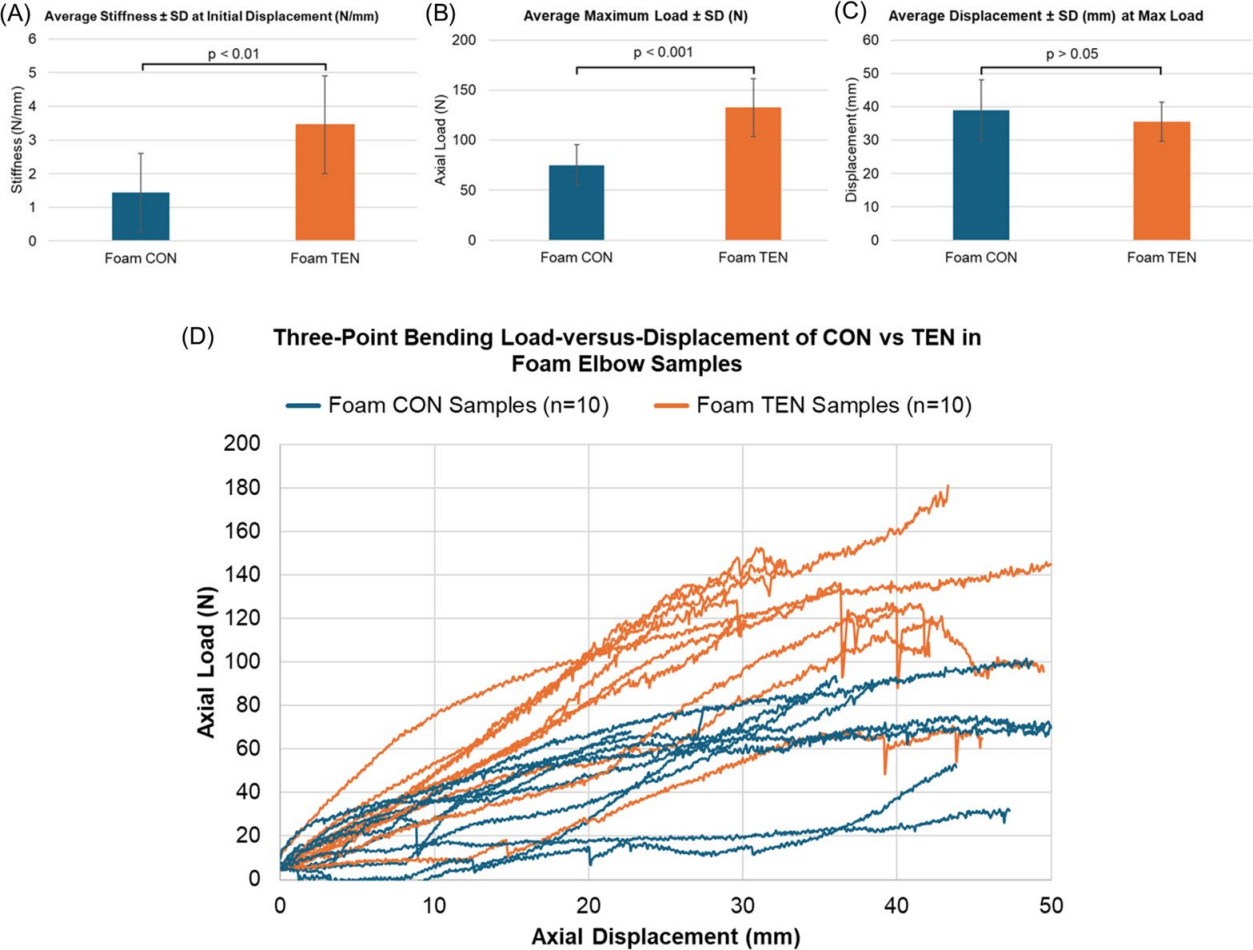
Results of the static three‐point bending of CON versus TEN device in foam elbow samples (*n* = 10 each). (A) Average ± SD of stiffness (N/mm) of CON versus TEN devices at the initial displacement of the elastic region of the load–displacement curves (*p* = 0.003). (B) Average ± SD of maximum load (N) of CON versus TEN devices when static three‐point bending was performed (*p* = 0.0001). (C) Average ± SD of displacement (mm) when the maximum axial load of CON versus TEN devices was reached (*p* = 0.3183). (D) Load–displacement curves of CON versus TEN devices of the static three‐point bending tests at 1 mm/s rate with a maximum displacement of 50 mm.

The elastic modulus was significantly higher for TEN devices (Figure 2A,D). At initial displacement, mean ± SD of stiffness values was 1.44 ± 1.17 and 3.46 ± 1.45 N/mm for CON and TEN devices, respectively (*p* = 0.0029). The plotted load–displacement curves for all samples also consistently showed that the TEN samples had higher force magnitudes than CON samples (Figure 2D). For CON and TEN samples respectively, mean ± SD values of the maximum forces were 75.02 ± 20.28 and 132.60 ± 28.82 N (Figure 2B, *p* = 0.0005); however, there were no statistically significant differences in the displacements where the peak forces were reached with mean ± SD of 39.03 ± 9.05 and 35.54 ± 5.80 mm for CON and TEN devices, respectively (Figure 2C, *p* = 0.2176).

**FIGURE 3:**
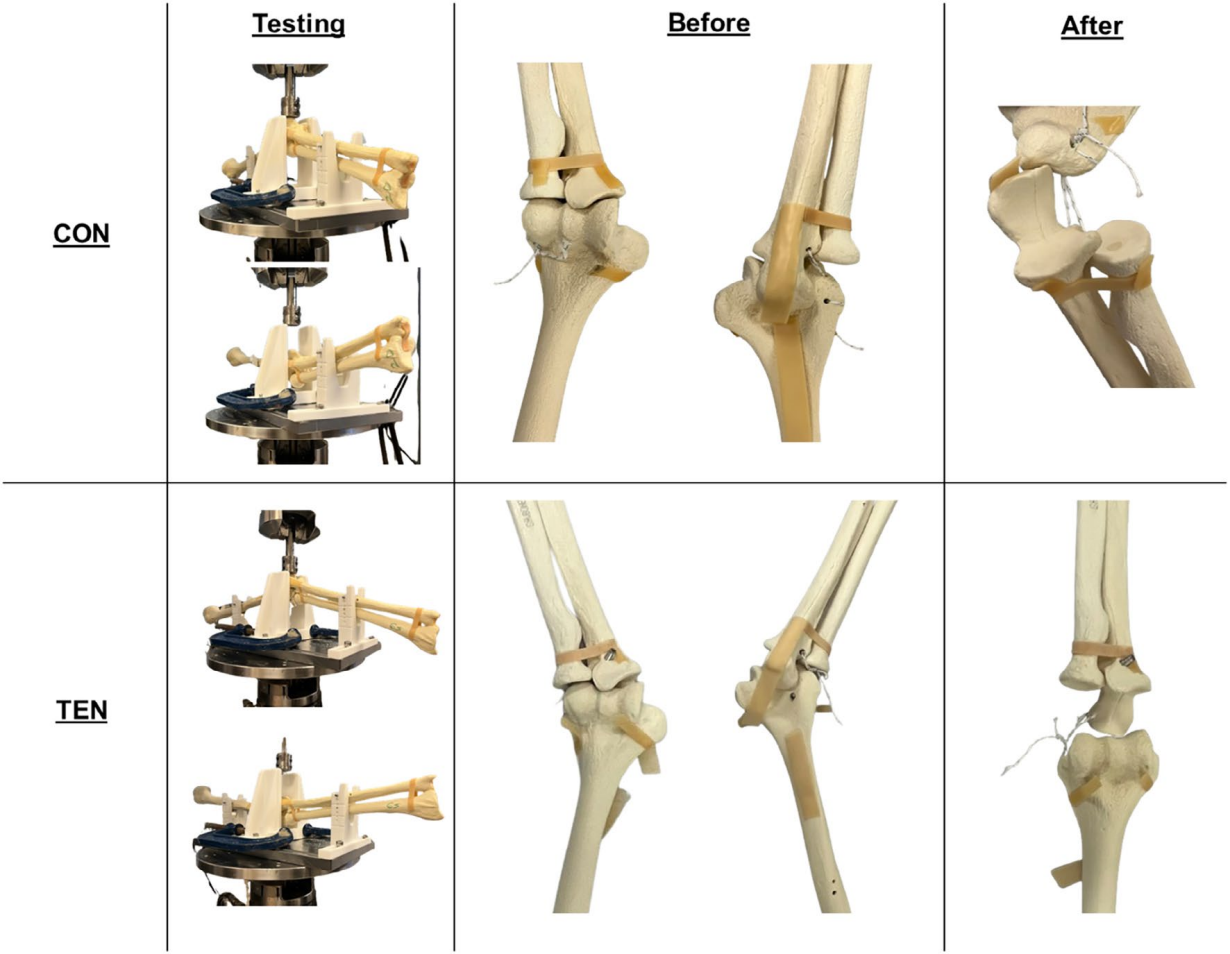
Representative images of the static three‐point bending test setup and the samples of the two groups with foam bone before and after testing.

After the static three‐point bending tests, all 10 CON samples failed with the suture‐foam interfaces irreversibly stretched (Table 1, Figure 3, top). This indicated that the fixation was permanently yielded. Further inspection of the suture‐foam interface revealed plastic deformation at high local stress sutures turning corners with foam bone compaction, thus suggesting that the fixation method cannot be further improved by changing the suture material. Three of the TEN samples had the suture‐implant‐foam interfaces irreversibly stretched, one sample had the suture nearly break, while the majority of the samples, six of them, had the sutures break at the maximum load values (Table 1, Figure 3, bottom). Therefore, this indicated that the foam bone‐implant or suture‐implant interfaces were sufficiently resistant to yielding. Fixation could then be further enhanced by selecting a stronger suture material aside from FiberWire No. 5 [35, 36].

**TABLE 1:** Quantitative (e.g., stiffness, maximum load, and displacement at maximum load) and qualitative (e.g., failure mode) characteristics of the individual CON versus TEN samples (*n* = 10 each group) in foam elbow bone.

Samples	Stiffness (N/mm)	Max load (N)	Displacement (mm)	Failure mode
*Conventional docking samples (CON) in foam elbow bone*				
CON_Sample_01	3.64	75.25	43.80	Yield deformity at suture holes with irreversible elongation of suture
CON_Sample_02	0.41	72.75	48.93	Yield deformity at suture holes with irreversible elongation of suture
CON_Sample_03	1.00	53.04	43.74	Yield deformity at suture holes with irreversible elongation of suture
CON_Sample_04	2.50	101.59	48.38	Yield deformity at suture holes with irreversible elongation of suture
CON_Sample_05	0.36	32.60	47.19	Yield deformity at suture holes with irreversible elongation of suture
CON_Sample_06	1.69	79.38	27.49	Yield deformity at suture holes with irreversible elongation of suture
CON_Sample_07	2.63	68.16	22.66	Yield deformity at suture holes with irreversible elongation of suture
CON_Sample_08	1.44	89.55	38.60	Yield deformity at suture holes with irreversible elongation of suture
CON_Sample_09	0.65	84.46	33.52	Yield deformity at suture holes with irreversible elongation of suture
CON_Sample_10	0.06	93.48	36.01	Yield deformity at suture holes with irreversible elongation of suture
Average (*n* = 10)	1.44	75.02	39.03	
SD (*n* = 10)	1.17	20.28	9.05	
*Tensegrity samples (TEN) in foam elbow bone*				
TEN_Sample_01	3.88	181.08	43.30	Suture ruptured
TEN_Sample_02	4.18	145.86	30.76	Yield deformity at screw hole and screw migration
TEN_Sample_03	4.10	136.52	35.98	Suture ruptured
TEN_Sample_04	0.65	70.67	43.59	Yield deformity at screw hole with irreversible elongation of suture
TEN_Sample_05	1.98	126.96	40.82	Yield deformity at screw hole with irreversible elongation of suture
TEN_Sample_06	5.61	146.90	32.39	Suture ruptured
TEN_Sample_07	4.93	152.30	30.88	Suture ruptured
TEN_Sample_08	2.63	114.21	39.30	Yield deformity at screw hole and screw migration
TEN_Sample_09	3.56	130.48	28.79	Suture ruptured
TEN_Sample_10	3.04	121.02	29.60	Suture ruptured
Average (*n* = 10)	3.46	132.60	35.54	
SD (*n* = 10)	1.45	28.82	5.80	

### 3.2 Performance in Cadaveric Bone Model

**FIGURE 4:**
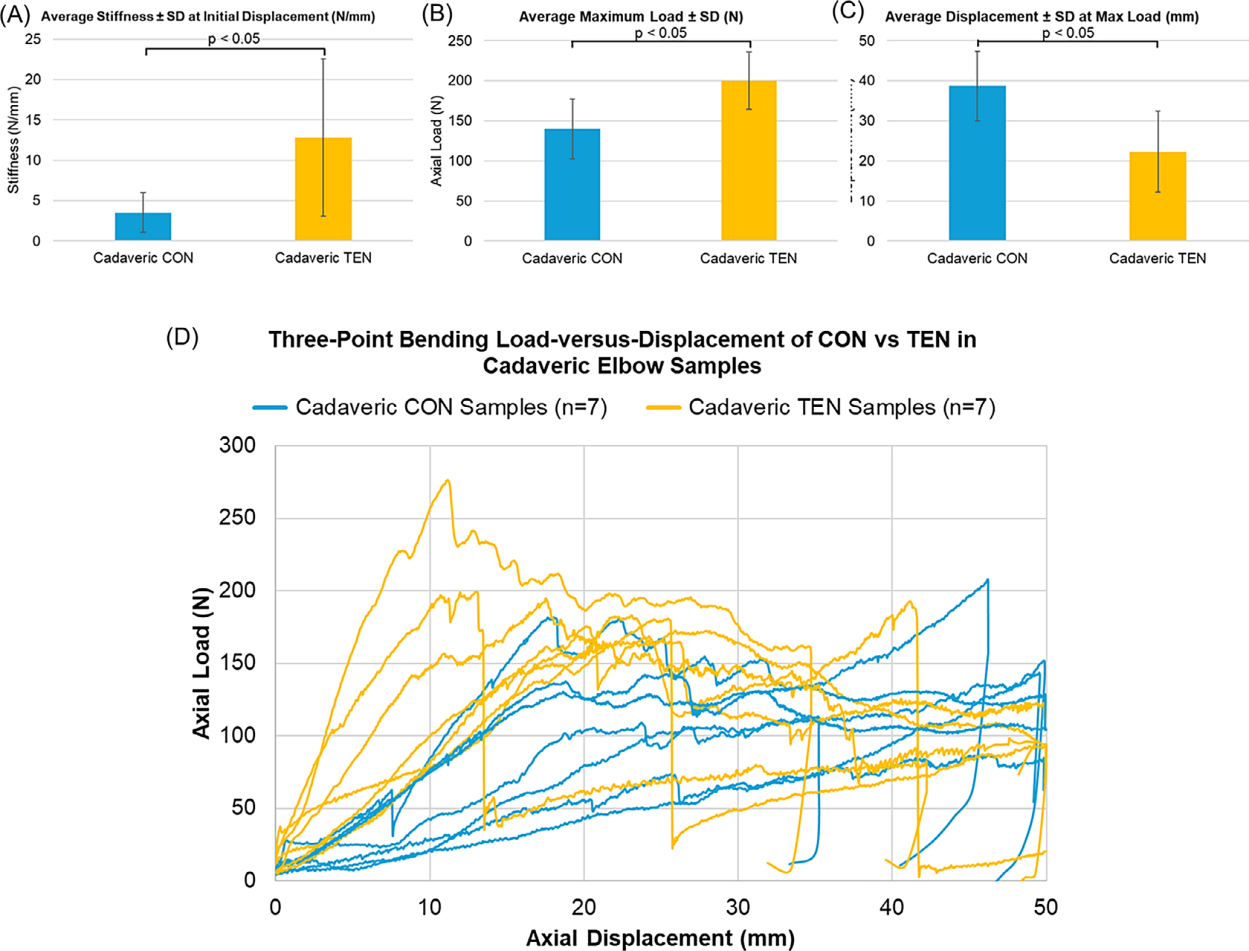
Results of the static three‐point bending of CON versus TEN device in cadaveric elbow samples (*n* = 7 each). (A) Average ± SD of stiffness (N/mm) of CON versus TEN devices at the initial displacement of the elastic region of the load–displacement curves (*p* = 0.0309). (B) Average ± SD of maximum load (N) of CON versus TEN devices when static three‐point bending was performed (*p* = 0.0099). (C) Average ± SD of displacement (mm) when the maximum axial load of CON versus TEN devices was reached (*p* = 0.0067). (D) Load–displacement curves of CON versus TEN devices of the static three‐point bending tests at 1 mm/s rate with a maximum displacement of 50 mm.

The results in the cadaveric bone model for the CON and TEN devices were generally consistent with the foam bone model; however, due to the variation of quality and performance in cadaveric bone samples, the SDs were much higher than the surrogate foam. Regardless, the differences in stiffness, maximum load, and displacements at maximum load results were still statistically significant. In consistency with the foam bone model, the elastic modulus was significantly higher for TEN devices (Figure 4A,D). At initial displacement, average ± SD of stiffness values was 3.53 ± 2.43 and 12.79 ± 9.73 N/mm for CON and TEN devices, respectively (*p* = 0.0111). The plotted load–displacement curves for all samples also consistently showed that the TEN samples had higher force magnitudes than CON samples (Figure 4D). For CON and TEN samples respectively, average ± SD values of the maximum forces were 140.11 ± 37.23 and 199.93 ± 35.89 N (Figure 4B, *p* = 0.0175). TEN devices had no significant differences in the displacements where the peak forces were reached with an average ± SD of 38.68 ± 8.64 and 22.31 ± 10.06 mm for CON and TEN devices, respectively (Figure 4C, *p* = 0.0111).

**FIGURE 5:**
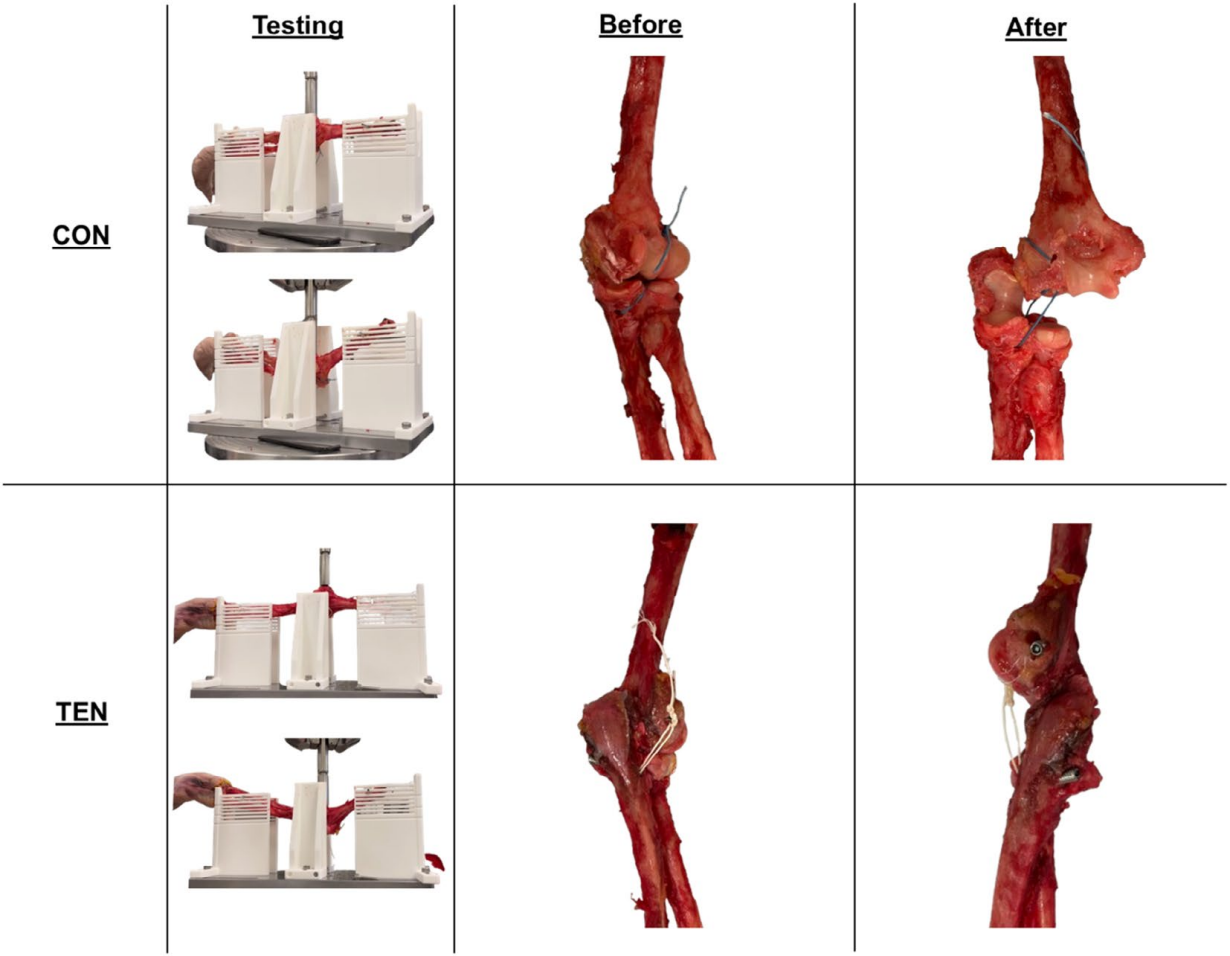
Representative images of the static three‐point bending test setup and the samples of the two groups with cadaveric bone before and after testing.

After the static three‐point bending tests, all seven CON samples failed with fixation loosening without suture rupture or obvious elongation (Table 2). Consistent with the CON surrogate bone samples, stress concentration in suture turning corners led to yield deformation in all seven samples. Meanwhile, four of the seven TEN samples had screw pull‐out as the bone fractured around it, and three TEN samples had the sutures ruptured at the maximum load. This indicated that bone quality was a major determining factor for fixation strength, which is enhanced by better stress distribution of the TEN devices (Table 2, Figure 5).

**TABLE 2:** Quantitative (e.g., stiffness, maximum load, and displacement at maximum load) and qualitative (e.g., failure mode) measures of the individual CON versus TEN samples (*n* = 7 each group) in cadaveric elbow bone.

Samples	Stiffness (N/mm)	Max load (N)	Displacement (mm)	failure mode
*Conventional docking samples (CON) in cadaveric elbow bone*				
CON_Sample_01	1.00	207.94	46.21	Fracture at suture hole with irreversible elongation of suture
CON_Sample_02	6.73	113.37	35.19	Fracture at suture hole with irreversible elongation of suture
CON_Sample_03	2.74	151.86	34.49	Fracture at suture hole with irreversible elongation of suture
CON_Sample_04	0.83	143.57	49.53	Fracture at suture hole with irreversible elongation of suture
CON_Sample_05	2.13	87.12	45.60	Fracture at suture hole with irreversible elongation of suture
CON_Sample_06	5.90	142.64	25.38	Bone impaction at suture hole with irreversible elongation of suture
CON_Sample_07	5.35	134.27	34.39	Bone impaction at suture hole with irreversible elongation of suture
Average (*n* = 7)	3.53	140.11	38.68	
SD (*n* = 7)	2.43	37.23	8.64	
*Tensegrity samples (TEN) in cadaveric elbow bone*				
TEN_Sample_01	30.21	276.52	11.16	Suture ruptured
TEN_Sample_02	20.77	199.38	13.07	Fracture and screw pull‐out
TEN_Sample_03	6.36	183.25	23.08	Fracture and screw pull‐out
TEN_Sample_04	3.56	192.76	41.16	Fracture and screw pull‐out
TEN_Sample_05	6.00	165.81	24.98	Fracture and screw pull‐out
TEN_Sample_06	7.87	180.72	25.42	Suture ruptured
TEN_Sample_07	14.79	201.08	17.30	Suture ruptured
Average (*n* = 10)	12.79	199.93	22.31	
SD (*n* = 7)	9.73	35.89	10.06	

### 3.3 Effect Size and Post Hoc Power Analysis

Median values with interquartile ranges (IQRs), Hodges–Lehmann median differences, common language effect sizes (CLESs), and rank‐biserial correlations were reported alongside *p*‐values to place emphasis on clinical rather than purely statistical significance (Table 3). Post hoc power calculations showed > 80% power for all but one parameter (displacement at maximum load in foam models), which reached 57%. These results indicate that despite the modest sample size, the study was sufficiently powered to detect large effects—common in tightly controlled biomechanical research—making type II errors unlikely. Cross‐model comparisons further showed consistent replication of stiffness and maximum load findings between foam and cadaveric models, although the foam model underestimated the stabilization benefit of the TEN devices observed in cadaveric specimens.

**TABLE 3:** Statistical analyses of biomechanical testing for CON versus TEN samples in foam and cadaveric elbow bone with their corresponding mean and standard deviation (SD), median, interquartile range (IQR), Hodges–Lehmann median difference, Mann–Whitney *U*, *p* values, common language effect size (CLES), and rank‐biserial correlation values.

Parameter	Group	Mean ± SD	Median (IQR)	Hodges–Lehmann median difference	Mann–Whitney *U* ^a^	*p*	CLES^b^	Rank‐biserial correlation^c^
*Foam bone model (n = 10 per group)*								
Stiffness (N/mm)	CON	1.44 ± 1.17	1.22 (0.41–2.50)	+2.20	12	0.0029	0.88	0.76
	TEN	3.46 ± 1.45	3.72 (2.63–4.18)					
Maximum load (N)	CON	75.02 ± 20.28	77.31 (68.16–89.55)	+57.25	7	0.0005	0.93	0.86
	TEN	132.60 ± 28.82	133.50 (121.02–146.90)					
Displacement at maximum load (mm)	CON	39.03 ± 9.05	41.17 (33.52–47.19)	−4.61	33	0.2176	0.57	0.34
	TEN	35.54 ± 5.80	34.19 (30.76–40.82)					
*Cadaveric model (n = 10 per group)*								
Stiffness (N/mm)	CON	3.53 ± 2.43	2.74 (1.00–5.90)	+5.53	5	0.0111	0.90	0.80
	TEN	12.79 ± 9.73	7.87 (6.00–20.77)					
Maximum load (N)	CON	140.11 ± 37.23	142.64 (113.37–151.89)	+55.81	6	0.0175	0.88	0.76
	TEN	199.93 ± 35.89	192.76 (180.72–201.08)					
Displacement at maximum load (mm)	CON	38.68 ± 8.64	35.19 (34.39–46.21)	−17.88	5	0.0111	0.90	0.80
	TEN	22.31 ± 10.06	23.08 (13.07–25.42)					

^a^ *U* values calculated referred to TEN versus CON study groups.

^b^ As *U* value was calculated as TEN group versus CON group, the effect size for a Mann–Whitney test can be calculated by using CLES, where CLES = *P*(*B* > *A*) = 1 − *U*/(*n*_1_· *n*_2_), where *U* is the Mann–Whitney *U* statistic and *n*_1_· *n*_2_ are the sample sizes of the two groups. CLES = 0.5 meant no effect or equal groups, CLES > 0.5 meant the TEN group was favored over the CON group and vice versa.

^c^ Rank‐bisserial correlation quantified the strength and direction of the relationship between two groups, *r* = 1–2 *U*/(*n*_1_· *n*_2_), where *U* is the Mann–Whitney *U* statistic and *n*_1_· *n*_2_ are sample sizes of the two groups. *r* = 0 meant no effect, *r* > 0 meant TEN group > CON group, ∣*r*∣ = 0.1 meant small effect, ∣*r*∣ = 0.3 meant medium effect, and ∣*r*∣ = 0.5 meant large effect.

## 4 Discussion

### 4.1 Principal Findings of This Study

**FIGURE 6:**
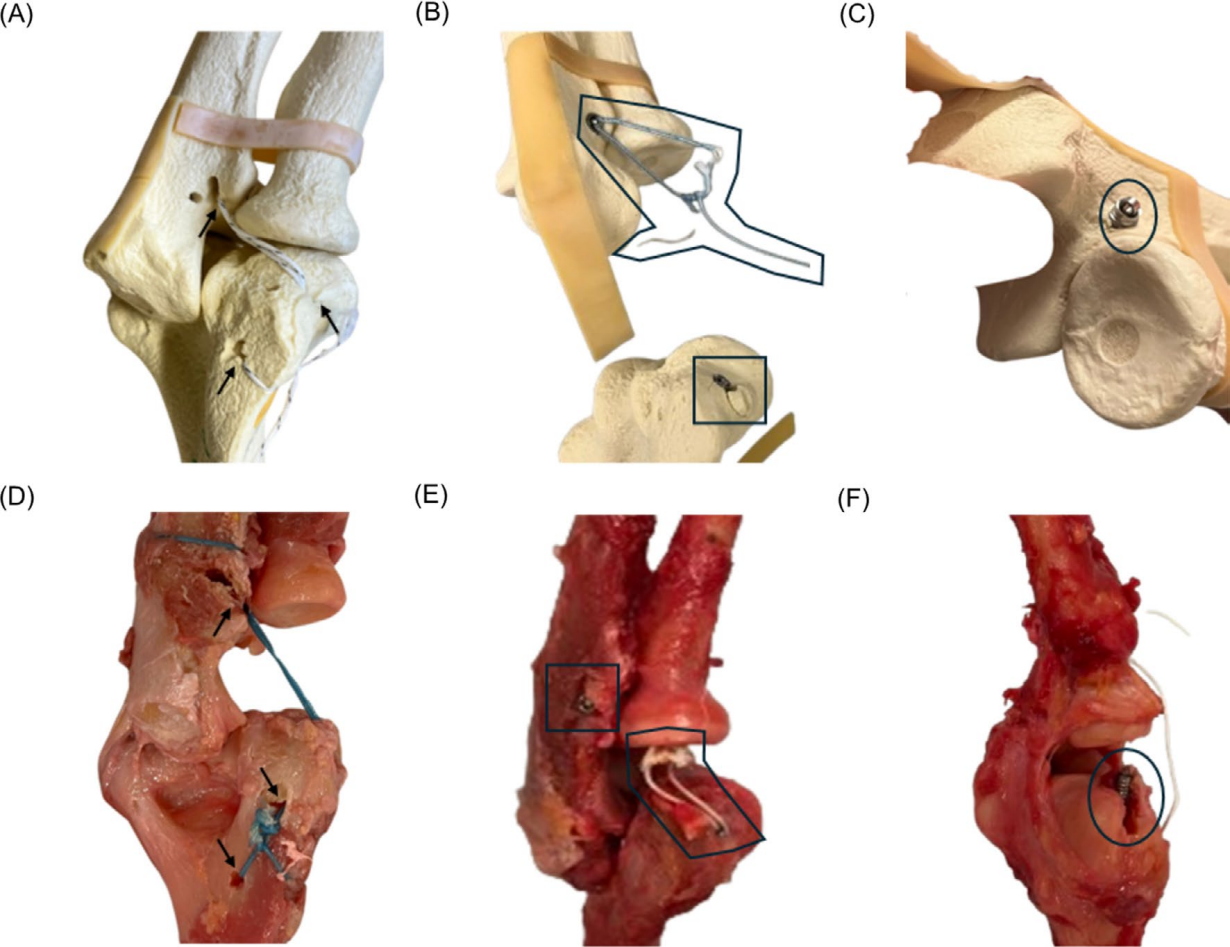
Close up of all types of failure mechanisms observed: (A) yielding at suture holes (shown with black arrows) with irreversible elongation of suture in a CON surrogate example; (B) suture rupture (outlined) and bone fracture (in rectangular box) in a TEN surrogate sample; (C) screw migration/pullout (circled) in a TEN surrogate sample; (D) yielding at suture holes (shown with black arrows) with irreversible elongation of suture in a CON cadaver specimen; (E) suture rupture (outlined) and bone fracture (in rectangular box) in a TEN cadaver specimen; (F) fracture and screw pull‐out (circled) in a TEN cadaver specimen.

In this proof‐of‐concept study, the use of a surrogate bone model with Sawbones was intentional to establish baseline mechanical behavior under reproducible and controlled conditions (e.g., homogeneous density and absence of biological variability). A published review paper has validated that synthetic whole‐bone models, such as humerus analogs, provide consistency while replicating human biomechanical properties [37]. Sawbones (Pacific Research Laboratories, Vashon, WA) has long been used as a bone surrogate in lieu of cadaveric bone for biomechanical testing [38, 39, 40]. Moreover, the latest fourth‐generation Sawbones which was used in this study, has been shown to have further improvement in replicating the biomechanical and failure characteristics of human bone [40, 41]. By using the Sawbones model, we can minimize the sample size and improve the generalizability of the study due to its consistent manufacturing of design and quality with interspecimen variability not exceeding 10% [42]. Thus, in this controlled environment, the TEN system demonstrated significantly superior stiffness and load‐to‐failure compared to the CON technique, thereby providing a clear baseline of its mechanical advantage (Figure 6).

### 4.2 Validation on Translational Potential of TEN Versus CON in Cadaveric Model

To confirm translational validity, the experiments were replicated in cadaveric models, which are more representative of in vivo conditions. The greater stiffness and maximum load of the TEN system, first established in the surrogate model, were confirmed in the biologically variable cadaveric setting. These findings suggested that the TEN devices provide better joint stabilization and higher resistance to loading forces compared to CON devices. Furthermore, a critical new finding emerged: the displacement at failure for TEN devices was significantly smaller than CON in the cadaveric model (22.31 ± 10.06 vs. 38.68 ± 8.64 mm, *p* = 0.0067), unlike in the surrogate model where they were comparable (35.54 ± 5.80 vs. 39.03 ± 9.05 mm, *p* = 0.3183). This discrepancy can be attributed to the differences in the specimens used. Cadaveric samples more closely simulate in vivo conditions and physiological responses, whereas foam bones offer greater consistency and homogeneity [26, 27]. In simpler and homogeneous models, differences between CON and TEN devices might not be as striking due to the lack of complex challenges. However, in more complex scenarios, the advantages of TEN devices, particularly their ability to distribute loading forces more effectively, become evident, resulting in smaller displacements and greater resistance to loading forces. These findings suggest that TEN devices are likely to perform better in real‐world applications.

The lower variability in displacement in TEN versus CON (SD: 5.80 vs. 9.05 mm in foam; 10.06 vs. 8.64 mm in cadaveric) suggested TEN provides more consistent graft fixation. This aligned with published literature of reduced “suture slippage” with tension‐adjustable system [43]. Moreover, TEN screw's tensionable design addresses two critical limitations of CON docking, namely (1) irreversible, surgeon‐dependent knot tensioning and (2) susceptibility to suture elongation and slippage. The TEN screw instrumentation system is designed to be simple and straightforward (Figure 1), with the use of a simple hex driver to drive in the screw and a generic T7 torx screwdriver to adjust the suture eyelet for suture insertion and tensioning, which can always be fine‐tuned post knot‐tying. The adjustable tensioning mechanism thus mitigates the risk of either over‐tightening or under‐tightening inherent in conventional knot‐tying.

### 4.3 Comparative Failure Mechanisms of TEN and CON Constructs

**FIGURE 7:**
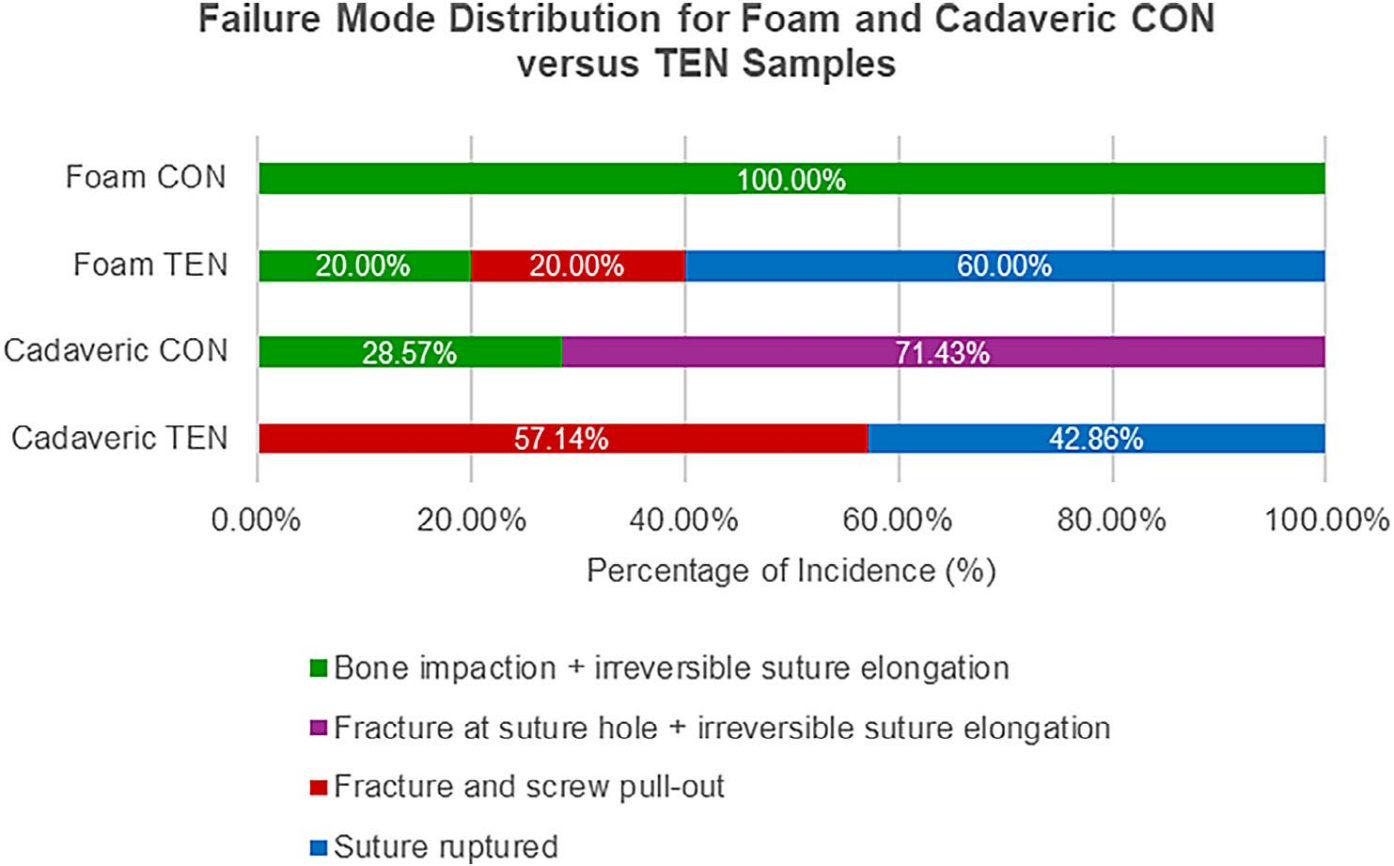
Failure mode distribution on CON and TEN devices in surrogate and cadaveric samples.

Regarding failure modes, the results were consistent across both the foam and cadaveric models (Figure 7). For CON devices, the two failure modes occurred concurrently, which were suture loosening due to irreversible suture stretching and yield deformation at suture sites (100% in the foam and cadaveric models). Under elevated mechanical loading in cadaveric models, CON devices had 28.6% (2/7) of specimens exhibiting bone impaction, while more showed progression to full bone fracture in 71.4% (5/7) of cases, along with permanent suture stretching. In contrast, TEN devices failed primarily due to suture rupture (60% in the foam model and 42.9% in the cadaveric model), irreversible yield deformation at the suture‐foam interface (40% in the foam model), and bone fracture with screw pull‐out (57.1% in the cadaveric model).

From these failure modes, several qualitative conclusions can be drawn. First, TEN devices are consistently stronger in mechanical strength and more durable than CON devices. The consistently high incidence of irreversible yield deformation in CON devices suggests reduced resistance to submaximal loads, leading to loosening and loss of tension. In contrast, TEN devices typically failed at peak load, indicating that the loading force was effectively distributed across the system until the FiberWire suture reached its limit. This implies that CON devices are more likely to fail over time due to daily use, potentially leading to recurrent elbow instability, whereas TEN devices are more likely to fail only under acute traumatic events, which are less common. Second, TEN devices demonstrate better bone preservation. In CON devices, bone fractures occurred at the suture sites, especially at turning corners, whereas in TEN devices, screw pull‐out was accompanied by bone fractures. Due to higher stress concentration, the CON configuration is more likely to fracture under lower loads. Thirdly, the tensegrity‐based system more closely mimics native ligament behavior. A study by Ciccotti et al. summarized the failure modes of native ligaments, reporting that 55% of samples failed due to bone avulsion and 45% due to mid‐substance tears [21]. Similarly, in the cadaveric model, 57.1% of TEN devices failed due to bone fracture, while 42.9% failed due to suture rupture. The suture rupture observed in TEN specimens when compared to permanent stretching in CON specimens, demonstrates effective load distribution to material failure, a response that closely mimics native ligament avulsion patterns. In clinical relevance, our measured failure loads (199.93 ± 35.89 N for TEN devices) are generally higher than usual physiological loads experienced during the process of normal activity or physical therapy. This similarity in failure modes suggests that TEN devices have a high potential to mimic native ligament function and restabilize the elbow joint instability in the early recovery phase.

Although more elastic materials reduce yield failure and irreversible deformation as seen in the CON technique, a rigid suture was deliberately chosen to increase the likelihood of yield failure as a worst‐case scenario. FiberWire, rather than autograft, was used for LUCL reconstruction to isolate fixation effectiveness from graft quality, providing uniform test conditions across samples. This approach enabled direct comparison of fixation methods under controlled variables and revealed suture rupture as the weakest link. FiberWire's material properties may influence bone deformation, and the use of more elastic grafts or weaker sutures could markedly alter construct stiffness. Ideally, suture material should combine high tensile strength with some elasticity for reversible elongation. While FiberTape offers superior strength [44], it could not be used due to incompatibility with the current anchor screw eyelets. Future studies with tendon grafts may refine the technique, though graft variability would reduce statistical power and increase the required sample size. Therefore, the present findings remain valid for comparing CON and TEN fixations under controlled laboratory conditions.

### 4.4 Limitations and Future Direction

There are some limitations in this study. First, the absence of a native ligament control group in the cadaveric model limits the ability to directly compare the performance of TEN devices with native tissue; however, the primary objective of this research, nevertheless, was to provide a direct comparison of the CON and novel TEN techniques, rather than comparing against native tissue function. Moreover, more recent studies have demonstrated comparable performance of UCL reconstruction docking with internal brace to its native ulnar collateral ligament [22, 45]. Therefore, while native controls would offer additional context, their absence does not hinder fair comparison of the two surgical methods under investigation. Preserving the lateral ligamentous complex, where feasible, would better reflect clinical practice and should be explored in future research. Incorporating a three‐point “hammock” structure—including the supinator crest and anterior capsule—could allow evaluation of CON and TEN techniques with greater biomechanical and anatomical relevance.

Secondly, there is a relatively wide age range of the cadaveric donors (range 79–94 years) with different quality of the specimens, such as bone density and other risk factors, that potentially influence the results with higher variability. Similar studies have also predominantly focused on similar demographics in older donors with mean ages of 72–79 years [18, 20, 21]. In the present study design, osteoporotic donors were chosen as a worst‐case scenario for implant fixation. Thus, the superiority of TEN devices observed even in this challenging model proves their robust mechanical properties and potential for clinical application.

Third, the relatively small sample sizes (20 for the surrogate model and 14 for the cadaveric model) may limit the statistical power of the study, although power analysis performed based on foam surrogate data showed that the sample sizes were adequate to generally achieve 80% power. To address the concern of small sample sizes, nonparametric Mann–Whitney tests were used considering the likelihood of nonnormal distribution and the higher risk of outliers skewing results. Despite the small sample sizes, post hoc power analysis including the large effect sizes and rank‐biserial effects confirmed that TEN screw generally exhibited greater load‐to‐failure resistance. These results should be interpreted as preliminary and to be validated in larger follow‐up studies.

Finally, the risk of screw pull‐out can be considered a limitation of the current TEN design especially in elderly and osteoporotic‐prone populations, as addressed in this study; however, it is also important to note that such failures also occurred at significantly higher loads (199.93 ± 35.89 N) in contrast to the loosening of sutures observed in CON specimens (140.11 ± 37.23 N). This points out that despite the TEN system possessing greater load‐carrying capability, there remains room for optimization of its bone‐implant interface.

## 5 Conclusion

Our findings consistently showed the superior performance of the TEN devices in terms of increase in mechanical strength, enhancement of suture‐screw‐bone fixation through appropriate junctional loading, and technical ease in terms of suture tensioning. The conventional docking technique requires precise techniques as control of suture tension and elimination of initial slack are inconsistent. The suture‐bone interfaces suffer from high stress concentration, and the system is prone to yield failure at significantly lower loads. Collectively, these findings warrant future validation of TEN devices through larger cohorts with diverse age groups, inclusion of native tissue controls, and clinical feasibility studies.

## Author Contributions

**Marilyn Janice Oentaryo:** investigation, methodology, formal analysis, resources, project administration, writing – original draft, visualization, data curation. **Tsz Ying Abby Yeung:** writing – original draft. **Christian Fang:** conceptualization, funding acquisition, supervision, project administration, writing – review and editing, methodology, investigation, resources.

## Funding

This study was cofunded by Hong Kong Innovation and Technology Fund grant ITS/304/20 and industry sponsorship by Lifespans Limited, Hong Kong. The authors declare no conflict of interest. The sponsors had no role in the design, execution, interpretation, or writing of the study.

## Conflicts of Interest

The authors declare no conflicts of interest.

## Supporting information


**Appendix Table S1:** Baseline demographics of the seven matched pairs of cadaveric sample with their randomized group allocations into conventional docking (CON) versus elbow tensegrity screw (TEN) devices. *M refers to specimen obtained from Medcure Inc., while S refers to specimen obtained from ScienceCare Inc.
