# Supplementary material for: Mechanical Evaluation of Suture Docking Method Versus Novel Tensegrity Suture Screw in Treating Varus Posterolateral Surrogate and Cadaveric Elbow Instability
Source: Orthop Surg. 2026 Jan 5;18(4):777–90. doi: 10.1111/os.70216 (PMC13056477; doi:10.1111/os.70216)
Supplement: Supplementary file 1 — Appendix Table S1: Baseline demographics of the seven matched pairs of cadaveric sample with their randomized group allocations into conventional docking (CON) versus elbow tensegrity screw (TEN) devices. *M refers to specimen obtained from Medcure Inc., while S refers to specimen obtained from ScienceCare Inc. [file OS-18-777-s001.docx]

**Appendix**

| Donor Number* | Age (years) | Height (in) | Weight (lb) | BMI (lb/in^2^) | Race | Sex | Side | Group |
| --- | --- | --- | --- | --- | --- | --- | --- | --- |
| M_01 | 94 | 62 | 102 | 18.65 | Caucasian | F | Left | TEN |
|  |  |  |  |  |  |  | Right | CON |
| M_02 | 92 | 60 | 154 | 30.07 | Caucasian | F | Left | TEN |
|  |  |  |  |  |  |  | Right | CON |
| M_03 | 86 | 62 | 124 | 22.68 | Caucasian | F | Left | CON |
|  |  |  |  |  |  |  | Right | TEN |
| S_01 | 90 | 58 | 76 | 15.88 | Caucasian | F | Left | TEN |
|  |  |  |  |  |  |  | Right | CON |
| S_02 | 92 | 61 | 127 | 23.99 | Caucasian | F | Left | CON |
|  |  |  |  |  |  |  | Right | TEN |
| S_03 | 83 | 65 | 76 | 12.65 | Caucasian | F | Left | CON |
|  |  |  |  |  |  |  | Right | TEN |
| S_04 | 79 | 63 | 94 | 16.65 | Caucasian | F | Left | TEN |
|  |  |  |  |  |  |  | Right | CON |

**Appendix Table 1.** Baseline demographics of the seven matched pairs of cadaveric sample with their randomized group allocations into conventional docking (CON) versus elbow tensegrity screw (TEN) devices. *M refers to specimen obtained from Medcure, Inc., while S refers to specimen obtained from ScienceCare, Inc.
